# S1P, Generated by Sphingosine Kinase 1, Negatively Affects Corneal Wound Healing Process by Activating TGF‐β/Smad Pathway

**DOI:** 10.1155/ancp/6404551

**Published:** 2026-07-21

**Authors:** Sandip K. Basu, Sarah E. Nicholas, Bradley P. Hambly, Nataliya Lenchik, Richard C. Grambergs, T. J. Hollingsworth, Amanda Prislovsky, Dimitrios Karamichos, Nawajes Mandal

**Affiliations:** ^1^ Department of Ophthalmology, University of Tennessee Health Science Center, Memphis 38163, Tennessee, USA, tennessee.edu; ^2^ North Texas Eye Research Institute; Department of Pharmaceutical Sciences, University of North Texas Health Science Center, Fort Worth 76107, Texas, USA, unthsc.edu; ^3^ Department of Family Medicine, Texas College of Osteopathic Medicine, University of North Texas Health Science Center, Fort Worth 76107, Texas, USA, unthsc.edu; ^4^ Department of Pharmaceutical Sciences, University of Tennessee Health Science Center, Memphis 38163, Tennessee, USA, tennessee.edu; ^5^ Department of Anatomy and Neurobiology, University of Tennessee Health Science Center, Memphis 38163, Tennessee, USA, tennessee.edu; ^6^ Memphis VA Medical Center, Memphis 38104, Tennessee, USA, va.gov

**Keywords:** corneal fibrosis, corneal injury, corneal wound healing, S1P signaling, S1P inhibitors, SMAD proteins, sphingosine kinase 1, TGFβ signaling

## Abstract

Abnormal healing of corneal injury can lead to corneal opacity, a leading cause of blindness. This process is extremely complex and involves precise interactions of multiple signaling pathways. A lack of understanding of these complex interactions provides a major challenge in developing therapeutic strategies. Both sphingosine‐1‐phosphate (S1P) and transforming growth factor beta (TGFβ) signaling have been associated with tissue fibrosis. The purpose of this study was to understand the interplay between S1P and TGFβ signaling in the process of corneal wound healing and fibrosis. Mice lacking the *Sphingosine kinase 1* gene (*Sphk1* ^−/−^) and their wildtype littermates were subjected to corneal injury by alkali burn. The progress of wound healing and the expression and activation of TGFβ signaling intermediates and profibrotic proteins were measured at different days postinjury (DPI). We observed that reduction of S1P signaling in *Sphk1* ^−/−^ mice induced the closure of corneal epithelial layer at a faster rate than its wildtype littermates following alkali burn. Reduced activation of profibrotic proteins Smad2 and 3, along with reduced expression of Smad4 and higher expression of antifibrotic protein Smad7, was also observed in the *Sphk1* ^−/−^ mice as compared to the wildtype littermates following corneal injury. Inhibition of S1P signaling by exogenous delivery of a small‐molecule inhibitor of sphingosine kinase 1 (SphK1) could accelerate corneal wound healing in similarly injured wildtype (*Sphk1* ^+/+^) mice. These results suggest that S1P signaling positively influences TGFβ signaling pathways that negatively affect corneal wound healing by delaying the process and inducing fibrotic development. Thus, inhibition of S1P signaling could be an effective therapeutic option to accelerate corneal wound healing and thereby prevent corneal fibrosis/scar formation.

## 1. Introduction

Corneal transparency, essential for the unimpeded transmission of light, is hindered due to corneal opacity or scars resulting from improper wound healing, which can result in vision loss. It is one of the leading causes of blindness worldwide and affects more than 1 million people in the United States [1, 2]. The corneal stroma is a collagen‐rich extracellular matrix (ECM) that features a precise and intricate arrangement of collagenous fibrils, essential for maintaining corneal transparency [3]. The corneal wound‐healing process often can disrupt stromal homeostasis by producing excess and disorganization of ECM components by activated fibroblasts and differentiated myofibroblasts, resulting in corneal scars [4]. The organization and regulation of ECM is a complex process involving the interactions of multiple signaling pathways [5–7]. One of the major challenges in identifying therapeutic targets and developing strategies that can ensure proper healing of corneal wounds and prevent corneal scar formation is the lack of a clear understanding of these complex interactions [8, 9].

The transforming growth factor β (TGFβ), along with its cognate receptors, is one of the most important players that regulate cellular physiology in ocular tissue, including differentiation and tissue repair [10–13]. TGFβ induces multiple growth factors that play an important role in the restoration of normal tissue following corneal injury [10, 14]. In mammals, three TGFβ isoforms, along with their receptors, are present in the cornea. TGFβ1 and TGFβ2 are known to disrupt normal stromal homeostasis, leading to fibrosis, whereas TGF‐β3 has been shown to have antifibrotic functions [15–19].

For the last two decades, several studies have established the role of sphingolipids in different cellular processes [20–25], with sphingosine‐1‐phosphate (S1P) being one of the major bioactive sphingolipids. At the cellular level, S1P is synthesized by phosphorylation of sphingosine by two kinases, sphingosine kinase 1 (SphK1) and 2 (SphK2). The two kinases have different subcellular localizations, thereby generating distinct pools of S1P with different functions [26]. SphK2 is localized inside the nucleus, generating the pool of S1P that regulates gene expression, while SphK1 is localized in the cytosol, generating S1P that functions as a second messenger inside the cell [27]. S1P is also known to be secreted out to act as an extracellular ligand for the five S1P receptors S1PR1‐5, initiating a signaling cascade that regulates cellular processes like proliferation, differentiation, and cell migration [28]. S1P has been shown to have a major role in tissue fibrosis [29, 30] of the skin [31, 32], liver [33, 34], heart [35, 36], and lung [37–39]. Studies from our group have implicated a role of S1P in corneal fibrosis and a crosstalk between S1P and TGFβ signaling pathways [40–42]. Here, we utilize an alkali burn model of corneal injury in wildtype (^+/+^) and global Sphk1 knockout (*Sphk1* ^−/−^) mouse models to delineate the role of S1P signaling in vivo in modulating the intermediates of the TGFβ pathway that are involved in corneal wound healing. We found that S1P and TGFβ signaling positively influence corneal scarring and fibrosis. Our findings will lead to a better understanding of the complex tissue healing process leading to scar formation following corneal injury.

## 2. Materials and Methods

### 2.1. Animal Care

10–15 weeks old *Sphk1* ^−/−^ mice in albino (BALB/c) background along with their wildtype littermates BALB/c (^+/+^) were utilized in this study. All the mice were born and raised in the University of Tennessee Health Science Center (UTHSC) vivarium, following its guidelines. The mice were maintained in dim (5–10 lx) cyclic light (12 h on/off) from birth. All procedures were performed according to the Association for Research in Vision and Ophthalmology Statement for the Use of Animals in Ophthalmic and Vision Research. The experimental procedures also followed the UTHSC Guidelines for Animals in Research, and the study protocol was reviewed and approved by the UTHSC Institutional Animal Care and Use Committee (IACUC Protocol Number 23‐425).

### 2.2. Animal Euthanasia

Animal euthanasia was performed following the UTHSC IACUC guidelines and Laboratory Animal Care Unit (LACU) SOP for rodent euthanasia. Briefly, the animals were placed in a covered plexiglass chamber fitted with a steady flow of compressed carbon dioxide (CO_2_) gas for 5 min, with the flow rate being determined by the LACU SOP. After 5 min, the flow of compressed gas was stopped, and the animals were kept submerged in CO_2_ gas for an additional 7 min. Then, the animals were visually observed for immobility and were taken out of the chamber. Cervical dislocation was performed as a secondary form of euthanasia before harvesting any tissues for further experiments.

### 2.3. Corneal Injury by Alkali Burn

The corneal injury by alkali burn was performed following a previously published procedure [43]. Briefly, the mice were anesthetized with an intraperitoneal (IP) injection of ketamine (100 mg/kg body weight) and xylazine (5 mg/kg body weight). A 0.5% proparacaine hydrochloride solution (Alcon Laboratories, TX) was then applied to the corneal surface as a preinjury analgesic. A 2 mm round piece of Whatman Number 1 filter paper, soaked in 0.5 N NaOH, was then applied to the central cornea of one eye for 20 s, followed by rinsing the eye with 0.9% sterile saline solution. Following injury, a topical antibiotic, erythromycin ophthalmic ointment 0.5% (Bausch & Lomb, NY), was applied to the burned cornea. Subcutaneous (SQ) injection of buprenorphine SR (0.6 µg/kg body weight) was used as a postinjury analgesic.

### 2.4. Corneal Imaging

Imaging of the ocular surface was performed using a fundus camera (Phoenix‐Micron Inc., OR) to characterize the corneal wound healing process following a previously published procedure [44]. Briefly, at different days postinjury (DPI), the mice were anesthetized as mentioned above, and one drop of 0.5% proparacaine hydrochloride was applied to each eye as an analgesic. A drop of 0.01% sodium fluorescein was applied to each eye, and the eyes were washed with 0.9% sterile saline solution after 20 s to remove the excess fluorescein. The external surface of the cornea was imaged using a green filter to visualize the fluorescein that was retained due to abrasions in the corneal surface. The relative injury area was determined by dividing the area of the corneal surface that has retained the fluorescein stain by the area of the total corneal surface.

### 2.5. mRNA Isolation and qRT‐PCR Analysis

Total RNA was extracted and purified from ^+/+^ and *Sphk1* ^−/−^ mouse whole corneas using the Invitrogen RNA isolation kit (Fisher Scientific) according to the manufacturer’s protocol. cDNAs were synthesized using the isolated RNA using SuperScript III First‐Strand Synthesis Mix (Invitrogen, CA) following the manufacturer’s protocol. Subsequently, qRT‐PCR analysis was performed using TaqMan Fast Advanced Master Mix (Applied Biosystems, CA), TaqMan gene expression primers (Table S1), and the cDNA using the QuantStudio 3 Real‐Time PCR System (Thermo Fisher Scientific, IL). The TGFβ signaling pathway genes were tested, while RPL19 and GAPDH were used as housekeeping genes. MS Excel and GraphPad Prism 9 were used to calculate the comparative threshold cycle (Ct) gene expression values. Each experiment was repeated at least four times.

### 2.6. Protein Isolation and Western Blot Analysis

Whole corneas from ^+/+^ and *Sphk1* ^−/−^ mice were homogenized with RIPA lysis buffer along with protease inhibitors for 2 min, incubated on ice for 5 min, and homogenized again for 3 min. The samples were then incubated at 4°C overnight and centrifuged at 12,000 RPM for 15 min at 4°C. The supernatant containing cellular proteins was collected and quantified using the Pierce BCA Protein Assay Kit (Thermo Fisher Scientific, MA). The cellular proteins were separated using Novex 4%–20% Tris‐glycine denaturing gels (Life Technologies) and subjected to a series of primary antibodies (Table S2) following standard Western blot procedures [41]. Membranes were imaged using an iBright 1500 FL imaging system (Thermo Fisher Scientific, MA), and the images were analyzed using iBright analysis software. All target values were normalized to the expression of β‐actin. Each experiment was repeated at least five times.

### 2.7. Treatment With SPHK *I*_2_ Following Corneal Injury

To assess the role of SphK1 inhibitor in corneal fibrosis, the ^+/+^ mice were subjected to alkali burn as described above. However, to induce fibrosis, a more severe corneal wounding is achieved by using 1.0 N NaOH for the alkali burn. From the next day, the mice started receiving eye drops of 0.1% SPHK *I*_2_ (MedChemExpress, NJ) dissolved in sterile water with 25% DMSO (v/v) on the right eye, while the left eye received the vehicle (25% v/v DMSO in sterile water) twice a day for 14 days. At 35 DPI, the mice were euthanized, and their eyes were enucleated. The corneas were then subjected to whole‐mount immunohistochemistry to assess the level of fibrosis.

### 2.8. Immunohistochemistry

The immunohistochemistry of the corneas was done following a previously published procedure [45]. Following enucleation, the eyes were fixed in 4% paraformaldehyde (PFA) for 10 min, and the cornea was dissected along the limbus. The corneas were fixed in 4% PFA for an additional 10 min and then permeabilized in 1% Triton X‐100 in PBS for 2–3 days. Corneas were blocked in 10% horse serum (HS) and incubated overnight at 4°C with a Cyanine3 (Cy3) dye–conjugated antialpha smooth muscle actin (α‐SMA) antibody (Sigma–Aldrich, MO). Following washing, the corneas were stained with DAPI (1 µg/mL), flat‐mounted on a slide in 50% glycerol mounting media, covered by a coverslip, and sealed with nail polish. The entire corneas were imaged using a Lionheart FX imager (BioTek Instruments) and analyzed using ImageJ software (NIH, MD). The confocal images of the corneas were captured using a Zeiss LSM 900 microscope.

### 2.9. Histopathological Characterization of Corneal Injury

The histopathological characterization of the cornea was done at 3 and 35 DPI. Mice of both genotypes were injured using either 0.5 N or 1.0 N NaOH, following the procedure described above. Uninjured and injured mice from both genotypes were euthanized at 3 and 35 DPI. The eyes were enucleated and fixed by Prefer fixative (Anatech Ltd., MI), processed for paraffin‐embedded histology, cut into 10‐micron sections using a rotary microtome (Epredia HM 325, Fisher Scientific), and stained by H&E following published protocols [46]. The sections were imaged using an Eclipse 80i fluorescence microscope (Nikon, NY). Three mice of both genders were used for each genotype and each treatment group (3 and 35 DPI).

### 2.10. Statistical Analysis

All statistics were done using GraphPad Prism 10.2 analysis software (GraphPad Software, CA). Outlying data points were evaluated using a ROUT test (*Q* = 1%) and removed from the dataset if they were identified as outliers. A comparison between two genotypes was done using a mixed‐fitting model of two‐way ANOVA with Bonferroni’s multiple comparison test correction. A comparison was made between different DPIs within the same genotype using a mixed‐fitting model of two‐way ANOVA with Dunnett’s multiple comparison test correction. A comparison between the two groups was made using the Student’s *t*‐test. A *p*‐value of <0.05 was considered significant for all comparisons.

## 3. Results

### 3.1. Reduced S1P Signaling Accelerates the Epithelium Closure of Corneal Wound

**Figure 1:**
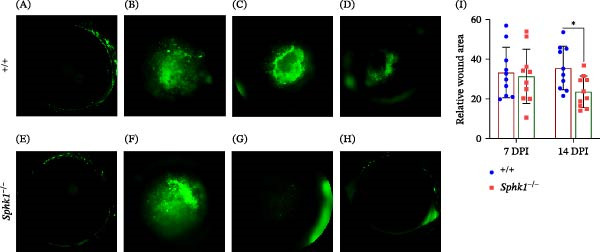
S1P signaling delays corneal wound healing postinjury. Representative longitudinal images of ^+/+^ and *Sphk1* ^−/−^ mice corneas showing fluorescein staining from the two groups’ uninjured (A, E) and injured corneas at 7 DPI (B, F), 14 DPI (C, G), and 21 DPI (D, H) showing the progression of corneal wound healing postinjury. (I) Quantitative analysis of relative wound area in square pixels from ^+/+^ and *Sphk1* ^−/−^ mice at 7 and 14 DPI (values represent mean ± SEM, *n* = 8 for each group, each genotype,  ^∗^ = *p* < 0.05).

Littermates from both ^+/+^ and *Sphk1* ^−/−^ mice were subjected to corneal injury by alkali burn, and the wound healing was monitored at different DPI by staining the cornea with sodium fluorescein that detects areas of epithelial deficiency as a result of corneal injury [47]. We did not observe any staining of the corneal surface of the uninjured mice from either of the genotypes, indicating an unblemished cornea (Figure 1A,E). At 7 DPI, the wounded area of the cornea between ^+/+^ and *Sphk1* ^−/−^ mice appears to be similar (Figure 1B,F). However, at 14 DPI, the wound in the *Sphk1* ^−/−^ mice appears to have considerably healed, whereas in the ^+/+^ mice, it remains similar to what was observed at 7 DPI (Figure 1C,G). By 21 DPI, the wound in *Sphk1* ^−/−^ mice appears to be completely healed, whereas there is still some corneal abrasion observed in the ^+/+^ mice, indicating a delayed wound healing process (Figure 1D,H). Quantitative analysis indicates that at 7 DPI, the wound area relative to the whole cornea surface area was similar between the two genotypes; however, at 14 DPI, we observed a significant reduction in the relative wound area in the *Sphk1* ^−/−^ mice compared to their wildtype littermates (Figure 1I).

### 3.2. Histological Characterization of Corneal Injury Following Alkali Burn

**Figure 2:**
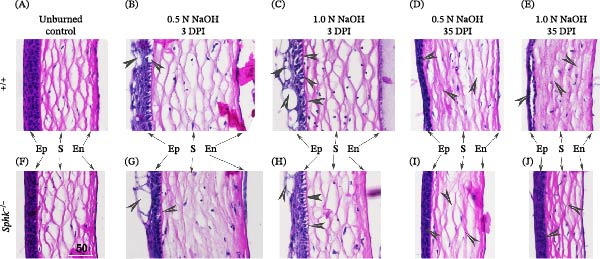
Histological characterization of corneal wound following corneal burn. Representative H&E‐stained images from ^+/+^ mice showing uninjured corneas (A) and injured with either 0.5 N NaOH at 3 DPI (B) and 35 DPI (D) or 1.0 N NaOH at 3 DPI (C) and 35 DPI (E). Representative corneal images from *Sphk1* ^−/−^ mice showing uninjured (F) along with corneas injured with 0.5N NaOH at 3 DPI (G) and 35 DPI (I) or 1.0 N NaOH at 3 DPI (H) and 35 DPI (J) showing the corneal structure following injury. The eyes were enucleated at the representative time post‐injury for both the genotypes and the injury conditions. Arrowheads at the different images marked the epithelium blistering, detachment of the epithelial layer from the stromal layer, and disorganization of epithelium and stroma following injury. Arrows are showing Ep (epithelium), S (stroma), and En (endothelium). The scale bar is 50 microns.

To compare corneal wound healing between ^+/+^ and *Sphk1* ^−/−^ mice, we performed histological analysis of unburned corneas and burned corneas at 3 and 35 DPI (Figure 2). Following injury with 0.5 N NaOH, both genotypes exhibited similar acute responses at 3 DPI, characterized by partial epithelial erosion and surface blistering (Figure 2B,G; arrowheads). By 35 DPI, epithelia had healed in both groups; in ^+/+^ mice, the epithelium was compressed to two cell layers with stromal disorganization (Figure 2D; arrowheads), whereas *Sphk1* ^−/−^ corneas resembled unburned controls with at least three epithelial layers and restored stromal organization (Figure 2I; arrowheads).

With 1.0 N NaOH, the acute response was more severe in both genotypes, showing complete epithelial disorganization, blistering, and Bowman’s membrane damage (Figure 2C,H; arrowheads). At 35 DPI, ^+/+^ mice displayed healed blisters but loss of epithelial layers, segmentation, and stromal disorganization (Figure 2E; arrowheads). In contrast, *Sphk1* ^−/−^ mice showed complete epithelial restoration, resembling unburned corneas without detachment or stromal disruption (Figure 2J; arrowheads).

### 3.3. S1P Signaling Regulates the Expression of TGFβ Signaling Intermediates Involved in Corneal Wound Healing

The physiological corneal wound‐healing process and its pathological counterpart that ultimately results in corneal fibrosis both involve the activation of canonical and noncanonical TGFβ pathways. To understand whether S1P signaling plays any role in the expression and activation of those intermediates, we performed qRT‐PCR with mRNA isolated from the uninjured and injured corneas at different DPI from both ^+/+^ and *Sphk1* ^−/−^ mice.

**Figure 3:**
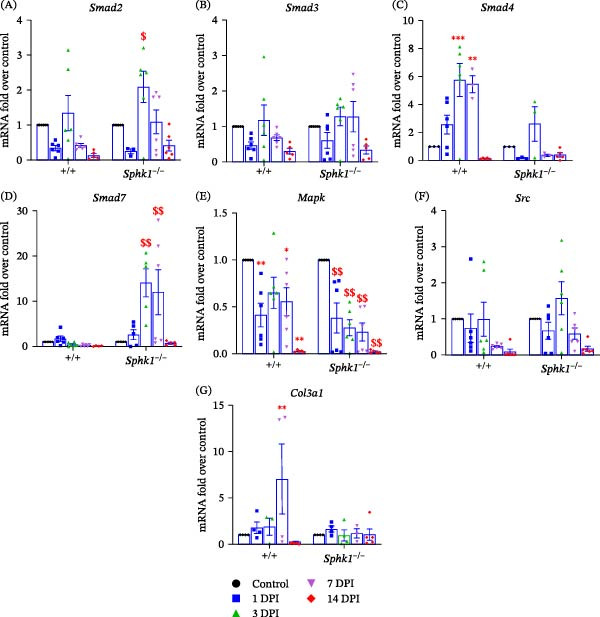
S1P signaling regulates expression of signaling intermediates involved in corneal wound healing process. The relative gene expression was determined by qRT‐PCR from uninjured control and injured corneas at different DPI of ^+/+^ and *Sphk1* ^−/−^ mice showing the expression pattern of *Smad2* (A), *Smad3* (B), *Smad4* (C), *Smad7* (D), *Mapk* (E), *Src* (F) and *Col3a1* (G). The fold change in expression at different DPIs of each genotype was represented compared to uninjured control of the same genotype. The  ^∗^ represents significance in ^+/+^ mice at respective DPIs compared to the uninjured control with  ^∗^ = *p* < 0.05,  ^∗∗^ = *p* < 0.01, and  ^∗∗∗^ = *p* < 0.001. The $ represents significance in *Sphk1* ^−/−^ mice at respective DPIs compared to the uninjured control with $ = *p* < 0.05 and $$ = *p* < 0.01 (values represent mean ± SEM, *n* = 6 for each group, each genotype).

For the canonical pathway, *Smad2* and *Smad3* showed similar overall trends with modest (≤1.5‐fold) changes (Figure 3A,B). In ^+/+^ mice, neither gene was significantly altered, but in Sphk1^−/−^ mice, although *Smad3* was unchanged, we observed a significant elevation of *Smad2* expression at 3 DPI (Figure 3A,B). *Smad4* expression in ^+/+^ mice increased sharply, peaking at ~6‐fold at 3 DPI before returning to baseline by 14 DPI, whereas no significant change was observed in *Sphk1* ^−/−^ mice (Figure 3C). Conversely, *Smad7* expression remained unchanged in ^+/+^ mice but was strongly induced (~10‐fold) at 3 and 7 DPI in *Sphk1* ^−/−^ mice (Figure 3D).

**Figure 4:**
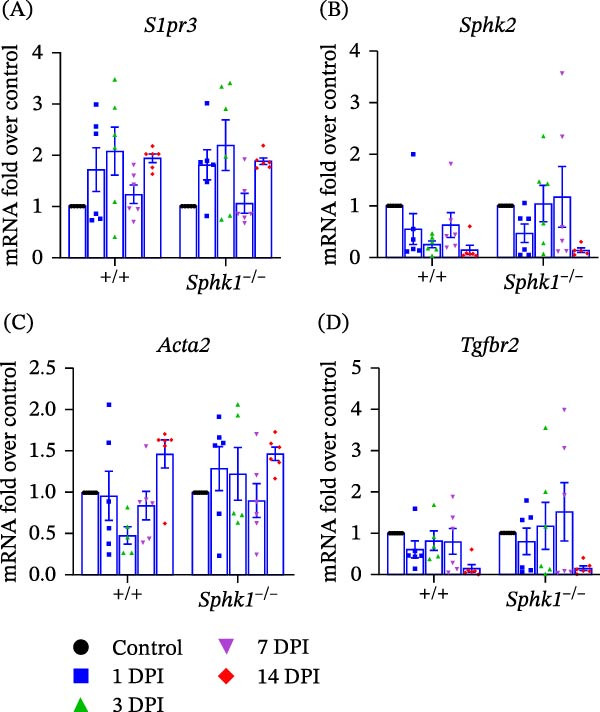
Expression of S1P and candidate genes of fibrotic pathway in mouse corneas. The relative gene expression of the S1P signaling pathway and fibrotic markers was determined by qRT‐PCR from uninjured control and injured corneas at different DPIs of ^+/+^ and *Sphk1* ^−/−^ mice showing expression patterns of *S1pr3* (A), *Sphk2* (B), *Acta2* (C) and *Tgfbr2* (D). The fold change in expression at different DPIs of each genotype was represented compared to uninjured control of the same genotype. No significant difference in expression was observed in either of the genotypes at different DPIs (values represent mean ± SEM, *n* = 6 for each group, each genotype).

In the noncanonical pathway, *Mapk1* and *Mapk3* were significantly reduced at all time points in both genotypes, while *Src* remained unchanged (Figure 3E,F). Among the downstream targets of TGFβ pathway, *Col3a1* was significantly upregulated at 7 DPI in ^+/+^ but not in *Sphk1* ^−/−^ mice (Figure 3G). Genes related to S1P signaling (*S1pr3* and *Sphk2*) and other fibrotic markers (*Acta2* and *Tgfbr2*) were unaffected by injury in either genotype (Figure 4A–D).

### 3.4. Reduced S1P Signaling Attenuates the Activation of TGFβ Pathway Intermediates Following Corneal Injury

**Figure 5:**
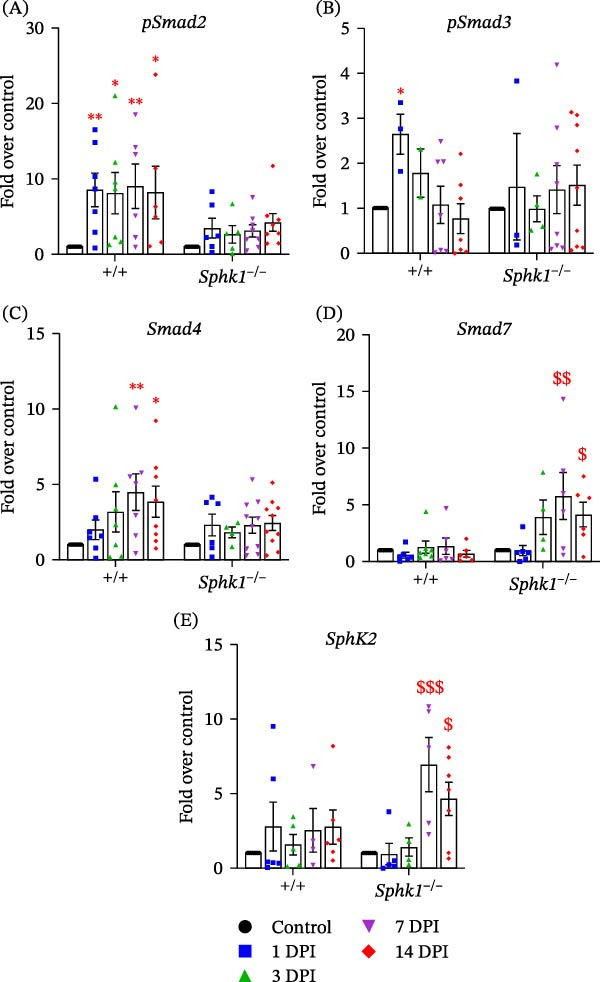
S1P signaling activates TGFβ signaling intermediates. Analysis of the TGFβ signaling pathway proteins from uninjured control and injured corneas at different DPIs of ^+/+^ and *Sphk1* ^−/−^ mice showing the relative expression and activation levels of pSmad2 (A), pSmad3 (B), Smad4 (C), Smad7 (D) and SphK2 (E). The fold change at different DPIs of each genotype was represented compared to uninjured control of the same genotype. The  ^∗^ represents significance in ^+/+^ mice at respective DPIs compared to the uninjured control with  ^∗^ = *p* < 0.05 and  ^∗∗^ = *p* < 0.01. The $ represents significance in *Sphk1* ^−/−^ mice at respective DPIs compared to the uninjured control with $ = *p* < 0.05 and $$ = *p* < 0.01 (values represent mean ± SEM, *n* = 6 for each group, each genotype).

We assessed the activation of TGFβ signaling intermediates by western blotting using total cellular proteins isolated from uninjured and injured corneas of ^+/+^ and *Sphk1* ^−/−^ mice at different DPIs, as described in Section 2. Among the components of the canonical TGFβ signaling, we observed that while the phosphorylation status of Smad2 significantly increased over uninjured control at all the DPIs analyzed in the ^+/+^ mice, the activation was severely attenuated in the *Sphk1* ^−/−^ mice with no significant increase in the phosphorylated Smad2 level at any of the postinjury days (Figure 5A). Smad3 also showed a significant increase in the phosphorylation status at 1 DPI in the ^+/+^ mice, whereas no significant increase in phosphorylated Smad3 was observed at any time points in the *Sphk1* ^−/−^ mice over the respective uninjured controls (Figure 5B). Similar to the alterations observed in the mRNA levels earlier (Figure 3C), Smad4 protein expression was increased over uninjured control in the ^+/+^ mice at 7 and 14 DPIs, with the maximum increase (~4.5‐fold) observed at 7 DPI. However, the *Sphk1* ^−/−^ mice did not exhibit a significant increase in Smad4 protein level following injury compared to their uninjured littermates (Figure 5C). While no significant changes were observed in Smad7 expression in the ^*+/+*^ mice after corneal injury, its expression was significantly increased over uninjured controls in 7 and 14 DPI in the *Sphk1* ^−/−^ mice (Figure 5D).

**Figure 6:**
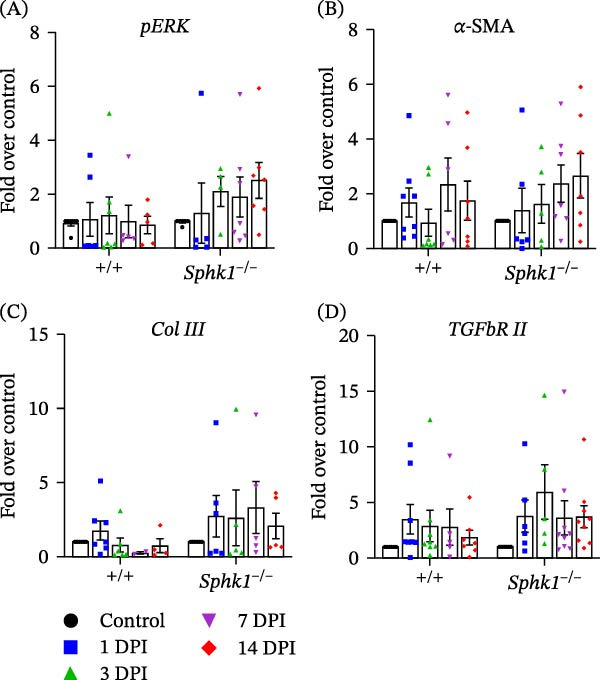
Protein expression of target genes of TGFβ signaling pathway. Evaluation of target genes of TGFβ signaling pathway from ^+/+^ and *Sphk1* ^−/−^ mice uninjured control and injured corneas at different DPIs showing the relative protein expression levels of pERK (A), aSMA (B), Col III (C) and TGFbRII (D). The fold change at different DPIs of each genotype was represented compared to uninjured control of the same genotype. No significant difference in expression was observed in either of the genotypes at different DPIs (values represent mean ± SEM, *n* = 6 for each group, each genotype).

The phosphorylation status of ERK1/2, the intermediate of the noncanonical pathway, has no significant changes in either genotype postinjury over the respective uninjured control (Figure 6A). Similarly, the protein levels of the transcriptional targets of the TGFβ pathway, α‐SMA, Col III, and TGFβ receptor II (TGFβRII), did not show any difference between the injured and uninjured corneas in either genotype (Figure 6B–D). However, we observed a significant increase in SphK2 protein levels at 7 and 14 DPI in Sphk1^−/−^ mice but not in their wildtype littermates (Figure 5E).

### 3.5. Topical Application of SPHK *I*_2_ Prevents Corneal Fibrosis Following Injury

**Figure 7:**
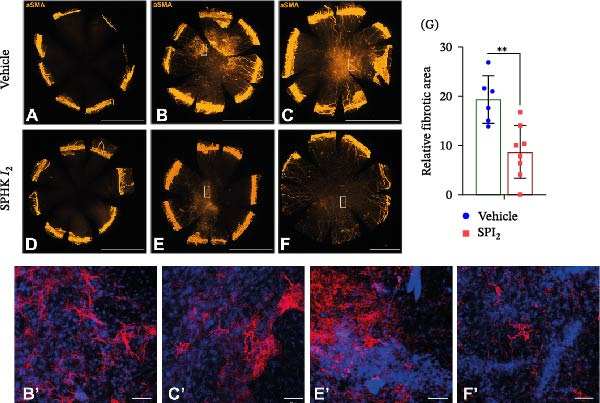
Inhibition of SK1 prevents corneal fibrosis following injury. Representative images of corneal flat mount showing α‐SMA staining from uninjured cornea receiving vehicle (A) or SPHK *I*_2_ eye drop (D) along with two individual injured corneas receiving vehicle (B, C) and SPHK *I*_2_ eye drop (E, F). Quantitative analysis (G) of the relative fibrotic area in square pixels compared to the whole cornea as measured by positive α‐SMA staining (values represent mean ± SEM, *n* = 8 for each group,  ^∗∗^ = *p* < 0.01). High‐magnification confocal images of panels B, C, E, and F were shown as insets B′, C′, E′, and F′, respectively.

Previous in vitro studies from our lab [41] and the data provided above indicate that S1P signaling has a positive influence in delaying the process of corneal wound healing after injury by activating TGFβ signaling intermediates that induce corneal fibrosis. To test this hypothesis, we used a selective small‐molecule inhibitor of SphK1 as a topical eyedrop following corneal injury in ^+/+^ mice and assessed fibrosis using immunohistochemistry for fibrotic markers, as described in Section 2. In the uninjured corneas, the fibrotic marker α‐SMA is completely absent, irrespective of the treatment with vehicle or SPHK *I*_2_ (Figure 7A,D). However, the eyes treated with SPKH *I*_2_ showed a reduced fibrotic area (as marked by the presence of α‐SMA) (Figure 7E,F) compared to the eyes receiving the vehicle as an eyedrop (Figure 7B,C). Quantitative analysis of the corneal images showed that the fibrotic area relative to the whole corneal surface is significantly reduced in the eyes receiving SPKH *I*_2_ as eyedrops compared to the eyes receiving vehicle following corneal injury (Figure 7G). High‐magnification confocal imaging shows α‐SMA–positive myofibroblasts in the central region of the burned corneas, more pronounced in the vehicle‐treated corneas than SPKH *I*_2_–treated corneas (Figure 7B′ inset of B,C′ inset of C, E′ inset of E, and F′ inset of F).

## 4. Discussion

In this study, we investigated the interplay between S1P and TGF‐β signaling in corneal wound healing following injury. Corneal wound healing involves multiple cellular physiological processes, including migration, apoptosis, differentiation, and ECM remodeling [48, 49]. Mild epithelial injury typically heals rapidly via limbal stem cell–mediated regeneration [48, 50], while deeper stromal injury triggers profibrotic cytokines such as TGFβ and PDGF. The signaling pathways initiated by these cytokines drive keratocyte differentiation into α‐SMA–positive myofibroblasts and disorganized ECM deposition, leading to corneal scarring [50]. The interaction between the S1P and TGFβ pathways in wound healing and tissue fibrosis has been documented in other tissues [29, 42, 51] and in the cornea or primary corneal fibroblasts in our previous studies [40, 52]. However, the complexities of these signaling pathways require system‐level studies, including the use of genetic mouse modes to fully decipher their signaling interplay. Here, using *Sphk1* ^−/−^ mice and subjecting them to alkali burn model, we investigated the role of S1P signaling in corneal wound healing and the repair process following injury.

Histological endpoints were induced with 1 N NaOH, while moderate injury (0.5 N NaOH) was used for biochemical analysis to preserve the tissue for assays. We used this model over debridement or keratotomy, as it is well standardized in our lab, which can be tuned to generate consistent, reproducible injury to the central cornea with desired levels of severity [43–45]. Our data clearly showed that S1P signaling delays the closure of the corneal epithelium and, thereby, the wound healing process. It also showed that loss of S1P signaling accelerates epithelial closure as *Sphk1* ^−/−^ mice healed faster than ^+/+^ mice after comparable injury (Figure 1). These data are consistent with earlier findings showing that loss of S1P signaling in *S1pr3* ^−/−^ mice improves wound healing following corneal injury by cauterization [53]. Histological analysis showed that the response to the injury and the healing process of both genotypes when injured by 0.5 N NaOH were very similar; however, the ^*+/+*^ mice showed some level of disorganization of the corneal epithelium layer and the collagen matrix in the stroma, which are early signs of fibrosis (Figure 2). In the case of a more severe injury (1.0 N NaOH), as expected, we observed a more severe effect, with complete disorganization of the corneal epithelial layer in both genotypes at the early phase (3 DPI). However, while the *Sphk1* ^−/−^ mice showed almost complete healing of the corneal wound by 35 DPI, the ^+/+^ mice showed severe disorganization of the collagen matrix and detachment of the epithelium layer from the stroma (Figure 2). Since stromal collagen architecture is critical for transparency, this disorganization likely hinders corneal transparency, leading to corneal scarring and fibrosis. The absence of such pathology in *Sphk1* ^−/−^ mice highlights the role of S1P in corneal wound healing, leading to scarring and fibrosis.

Another important factor contributing to the fibrotic process is the detachment of the epithelial layer from the stroma, which was also observed only in the ^+/+^ mice, further underscoring the positive role of S1P signaling in the pathological process of corneal wound healing following injury. It is pertinent to mention that even though no remarkable fibrosis was observed in injury sustained by 0.5 N NaOH, in either of the genotypes, the signaling intermediates that are known to positively affect the fibrotic process were more active in the ^+/+^ mice and were further confirmed by the biochemical analysis of the cornea (Figures 3–6).

It is worth mentioning that the strain of the mice used in this study (BALB/c) can also contribute to the lack of scarring following milder injury (0.5 N NaOH), as they have a lower tendency to develop α‐SMA–positive myofibroblasts [54]. Wound‐healing processes differ depending on the level of sustained injury [40], and it is possible that a different injury severity or a different strain or species of animals could modify the outcome. Our data also support the role of injury intensity in the development of detectable pathologies, as the same strain of mice (BALB/c) burned with a higher concentration of alkali (1 N NaOH) showed high expression of α‐SMA (Figure 7). So, the optimal concentration that would generate detectable pathologies without causing excessive tissue damage will vary based on the strain of the mice and the burning agent used. It will require implementing a standardization process to determine the optimal concentration to be used. Together, our results suggest that S1P delays epithelial closure and wound healing and positively influences the fibrotic process, likely through modulation of TGFβ signaling.

TGFβ signaling contributes to wound repair and fibrosis in various tissues, including the cornea, through both canonical Smad‐dependent and noncanonical pathways [14, 55]. The canonical pathway involves the Smad family of transcription regulators [56–58]. We observed higher Smad2/3 phosphorylation and Smad4 induction in ^+/+^ mice compared to *Sphk1* ^−/−^ (Figures 3C and 5A–C), indicating enhanced TGFβ signaling when S1P is intact. By contrast, Smad7 was significantly upregulated in *Sphk1* ^−/−^ but not in ^+/+^ mice (Figures 3D and 5D), consistent with inhibitory regulation of Smad activation. Thus, reduced Smad2/3 activity, lower Smad4, and higher Smad7 in *Sphk1* ^−/−^ corneas support a positive interaction between S1P and TGFβ that drives delayed wound repair. Smad2, 3, and 4 are known to positively influence the differentiation of primary equine corneal fibroblasts to myofibroblasts [59]. S1P stimulation induces phosphorylation of Smad2 and 3 in rat renal mesangial cells [60] and increases the expression of α‐SMA and induces transformation of human retinal pigment epithelium (RPE) cells to the profibrotic phenotype [61]. Additionally, the delivery of Smad7 to the corneal stroma can prevent corneal hazing in a rabbit model of corneal injury [62]. Our data reinforce these observations, highlighting S1P‐TGFβ crosstalk in corneal wound healing and fibrosis. Additionally, as previously published in our in vitro study [40], the current data suggest that the canonical TGFβ signaling pathway is the central driver of signal transmission in the cornea following injury. However, further detailed studies are necessary to clearly understand S1P’s role in Smad‐dependent and Smad‐independent signaling in the cornea following injury.

Candidate gene analysis revealed nonsignificant *S1pr3* upregulation after injury (Figure 4A). S1PR3‐S1P signaling axis has been shown to cooperate with the TGFβ‐Smad pathway, leading to fibrosis in multiple tissues [63]. We believe S1P signaling through S1PR3 leads to upregulation of TGFβ, which in turn signals through TGFβ receptor to induce SMAD proteins. Currently, studies using mice lacking S1PR3 are undergoing to test this hypothesis; however, further detailed studies are crucial to identify the S1P receptor(s) and to clarify the downstream intermediates that are involved in this process. Lipidomic analysis of the ^+/+^ and *Sphk1* ^−/−^ mice has shown an undetectable level of S1P in the cornea but a significantly lower level of S1P in the plasma in the *Sphk1* ^−/−^ mice [45]. It could be possible that the lower level of S1P in the *Sphk1* ^−/−^ mice attenuates the S1PR3‐S1P signaling axis even though there is a comparable level of S1PR3 expression in the cornea of that genotype. We have also observed a higher expression of the SphK2 protein in the *Sphk1* ^−/−^ mice (Figure 5E), which is not surprising since *Sphk1* ^−/−^ mice showed a higher expression of the *Sphk2* gene [45], probably as a compensatory mechanism to maintain the homeostatic level of S1P necessary for normal physiological functions. However, fibrotic protein markers α‐SMA and Col III were not significantly changed (Figure 6B,C). It is known that myofibroblast maturation and collagen deposition occur later in the healing process, leading to scar formation [16]. The severity of the injury, experimental timeline, and the strain of the mice utilized in this study could have influenced our observation. We observed the activation of fibrotic signaling intermediates and observed fibrosis in our model of a more severe injury (1 N NaOH) at a later time point (28–35 DPI) (Figure 7). A detailed study of the later time points postinjury is warranted to elucidate the expression patterns of these fibrotic markers in the corneal wound healing and fibrosis.

Corneal fibrosis remains a leading cause of blindness, with transplantation as the only treatment [2, 4]. Thus, identifying therapeutic candidates is urgently needed to combat this devastating pathology. Our data on the attenuation of S1P signaling by the SphK inhibitor SPHK *I*_2_ as an eye drop following injury showed significantly lower expression of α‐SMA in the cornea following injury, indicating a reduction in fibrosis (Figure 7). Inhibition of SphK1 and SphK2 by different classes of inhibitors, including amidine‐based, naphthalene‐based, and sphingoid‐guanidine‐based, has been studied in vitro and in vivo as a therapy for cancer, as well as for allergic and inflammatory diseases, cardiovascular and central nervous system diseases, and viral infections [64–67]. However, even with encouraging results in vitro, the systemic application of SphK inhibitors results in low potency, low specificity, and several off‐target effects [64, 65]. Thus, using such an inhibitor as a topical therapy is encouraging as it might reduce systemic toxicity. However, S1P plays an important role in physiological vascularization, and a topical therapy involving S1P inhibitors should be carefully chosen to avoid any possible side effects that hinder normal vascularization in the limbal region surrounding an avascular cornea.

## 5. Conclusion

The results from this study clearly established an interplay between S1P‐TGFβ signaling intermediates and their role in corneal wound healing following injury. In an animal model of corneal injury, we observed a positive cooperation between S1P signaling and TGFβ pathway that induces profibrotic proteins leading to the induction of α‐SMA essential for fibrotic development or scar formation. We also observed that reduction of S1P signaling was beneficial for the wound healing process in a preclinical mouse model. Our findings provide proof‐of‐concept that S1P inhibition could be a potential strategy to reduce corneal fibrosis and warrant further investigation into this pathway for therapeutic development.

## Author Contributions

Conceptualization: Nawajes Mandal and Dimitrios Karamichos. Data curation: Sandip K. Basu, Sarah E. Nicholas, Bradley P. Hambly, Nataliya Lenchik, Richard C. Grambergs, T. J. Hollingsworth, and Amanda Prislovsky. Formal analysis, validation, and methodology: Sandip K. Basu and Sarah E. Nicholas. Writing – original draft: Sandip K. Basu. Writing – review and editing: Sandip K. Basu, Sarah E. Nicholas, Nawajes Mandal, and Dimitrios Karamichos. Funding acquisition and supervision: Nawajes Mandal and Dimitrios Karamichos.

## Acknowledgments

The authors have nothing to report.

## Funding

This work has been supported by funding from the National Eye Institute (NEI) grant to Nawajes Mandal and Dimitrios Karamichos (Grant EY031316); NIDDK (NIH) grant to Nawajes Mandal (Grant DK128129); the Department of Veterans Affairs Biomedical Laboratory R&D (BLRD) grant to Nawajes Mandal (Grant BX004893); the US Department of Defense Office of the Congressionally Directed Medical Research Programs (CDMRP), Vision Research Program (VRP) grant to Nawajes Mandal (Grant W81XWH‐20‐1‐0900); Research to Prevent Blindness funding to Nawajes Mandal; and a Career Starter Research Grant from Knights Templar Eye Foundation to Sandip K. Basu.

## Conflicts of Interest

The authors declare no conflicts of interest.

## Supporting Information

Additional supporting information can be found online in the Supporting Information section.

## Supporting information


**Supporting Information** Table S1: Provides the list of TaqMan assays used in the RT‐qPCR with corresponding assay numbers. Table S2: Provides the list of antibodies used in the western blot experiments with corresponding catalog numbers.

## Data Availability

The data that support the findings of this study are available from the corresponding author upon reasonable request.
