# Supplementary material for: S1P, Generated by Sphingosine Kinase 1, Negatively Affects Corneal Wound Healing Process by Activating TGF‐β/Smad Pathway
Source: Anal Cell Pathol (Amst). 2026 Jul 21;2026:6404551. doi: 10.1155/ancp/6404551 (PMC13387310; doi:10.1155/ancp/6404551)
Supplement: Supplementary file 1 — Supporting Information Table S1: Provides the list of TaqMan assays used in the RT‐qPCR with corresponding assay numbers. Table S2: Provides the list of antibodies used in the western blot experiments with corresponding catalog numbers. [file ANCP-2026-6404551-s001.docx]

**Supplementary Table 1**

List of TaqMan assays used in RT-qPCR

| **Gene** | **Assay number** |
| --- | --- |
| *Smad2* | Mm00487530_m1 |
| *Smad3* | Mm01170760_m1 |
| *Smad4* | Mm03023996_m1 |
| *Smad7* | Mm00484742_m1 |
| *Erk1* | Mm00662375_g1 |
| *Erk2* | Mm00442479_m1 |
| *Src* | Mm00436785_m1 |
| *Col3A1 (Collagen III)* | Mm01254476_m1 |
| *S1PR3* | Mm02620181_s1 |
| *SphK2* | Mm00445021_m1 |
| *Acta2 (αSMA)* | Mm00725412_s1 |
| *Tgfbr2* | Mm03024091_m1 |
| *Rpl19* | Mm02601633_g1 |
| *Gapdh* | Mm00436864_m1 |

**Supplementary Table 2**

List of antibodies used in Western blots

| **Antibody** | **Catalogue Number** |
| --- | --- |
| pSMAD2 | ab53100 |
| pSMAD3 | ab52903 |
| SMAD4 | ab40759 |
| SMAD7 | ab226872 |
| SphK2 | ab37977 |
| α-SMA | ab5694 |
| Collagen III | ab184993 |
| TGF**-**βRII | ab61213 |
| β-actin | ab8227 |
